# Practices of Cancer Screening for Average-Risk Cancer Patients Among Primary Healthcare Center Physicians in Al-Qassim Region, Saudi Arabia

**DOI:** 10.7759/cureus.33829

**Published:** 2023-01-16

**Authors:** Moath Aljohani, Abdulrahman Alsaykhan, Ahmed Almutairi, Faisal Almadhi, Talal Alhawshani, Sael Almishrafi, Bader Alharbi

**Affiliations:** 1 Department of Family and Community Medicine, Unaizah College of Medicine and Medical Sciences, Qassim University, Unaizah, SAU

**Keywords:** qassim, practices, cancer, primary care, screening

## Abstract

Objective: Cancer screening programs exist in Saudi Arabia for some types of cancers. However, data on primary healthcare center (PHC) physicians’ practices in referring patients for screening tests or procedures remain unclear.

Methodology: A cross-sectional study was conducted with a self-reported survey that included 141 PHC physicians affiliated with the Ministry of Health in the Al-Qassim region of Saudi Arabia. The primary outcome was the practice of recommending to average-risk patients screening tests for different types of cancers including breast, colorectal, cervical, prostate, and lung, and testing if sociodemographic, specialty, job level, years of experience, a family history of cancer, and patients encountered per day affect their decisions. Secondary outcomes were the barriers perceived by physicians to recommending a screening test. p-value <0.05 was considered significant.

Results: The study included 141 respondents, of which 60.3% were males, and the mean age of the entire population was 35.7 ± 8.3 years. The rate of recommending cancer screening varied by the type of cancer, with screening for colorectal cancer being the most prominent (64.5%), followed by breast cancer (51.8%). Fear of finding cancer, poor patient compliance, and difficulty in scheduling the test were the most common patient, physician, and system-related barriers as perceived by PHC physicians. Male physicians were less likely to recommend patients for breast (0.10, 95%CI 0.04-0.23, p < 0.001) and cervical (0.26, 95%CI 0.08-0.78, p = 0.017) cancer screening. However, they were 3.74 times more likely to recommend prostate cancer screening (95%CI 1.20-11.68, p = 0.023) and 5.79 times more likely to request lung cancer screening (95%CI 1.27-26.39, p = 0.023).

Level of education, specialty, and being a senior physician were factors associated with cervical cancer screening. Physicians who work in non-general practice specialties were more likely to recommend cervical cancer screening than those who work in general practice (95%CI 0.04-0.48, p = 0.002). Senior physicians such as registrars/senior registrars and consultants were more likely to request or recommend a patient for breast cancer screening (2.85, 95%CI 1.11-7.35) and cervical cancer screening (6.35, 95%CI 2.10-019.19).

Conclusion: Screenings for colorectal and breast cancer were the commonly recommended screening tests. Patients' fear of finding cancer, poor patient compliance, and delays or difficulty in scheduling the procedures were the commonly identified barriers as perceived by physicians that influenced physician decisions in referring patients for cancer screening. Our findings suggest that cancer screening rates may be improved by educating individuals on the benefit of early detection of cancers and providing assurance for them with regard to the availability of effective treatments. More research is needed on ways to overcome the obstacles physicians encounter and the outcomes of these measures with regard to improved screening practices.

## Introduction

Cancer is one of the major causes of premature death and an increased number of deaths in the majority of countries this century. While cancer prevalence is increasing worldwide, its etiology is known to be multifactorial; some cancers are associated with the aging process and improved socioeconomic status [1].

In Saudi Arabia, cancer incidence showed an increasing trend in incidence with regard to thyroid, breast, colon, bladder, prostate, renal, and other cancers between 1990 and 2016. During the same period, there was also increased mortality due to these cancers. However, the proportion of mortalities due to cancers declined after the age of 70. The increase in the prevalence of different types of cancer can be attributed to wide-scale changes in the various aspects of social and economic status that have happened in the kingdom [2]. Screening is the process of applying a test or examination for the early detection of the disease or a certain health event, thereby halting the disease progression and preventing complications [3]. Though screening as a principle provides hope, it remains debatable in terms of cost in comparison to the expected benefit and harms and whether other types of cancer of public health importance require more attention [4]. Different modalities are being used in cancer screening based on the type of cancer and the availability of that modality; one of the most common is mammography for breast cancer screening. There is fair-quality proof that mammography decreases the risk of death by a range of 10-30% [5]. Similar ranges were noticed for colorectal cancer (CRC) screening through sigmoidoscopy or occult blood testing [6]. Based on a systematic review finding, the screening leads to a 35% (10-53%) decrease in risk of death when samples obtained through cervical swabbing are screened for cervical cancer [7]. The decision to screen or not for prostate cancer is conflicting [8]. For lung cancers, low-dose CT showed a decrease in the risk of mortality of 19%; however, it also showed high false positive rates, which might increase screening anxiety and warrant a review of the decision to screen or not compared to the expected benefits [9].

Screening practices among the Saudi population are low. Three-quarters of Saudis reported not going through routine screening despite the presence of multiple risk factors for chronic diseases; 60% of the population has some form of obesity, while a similar proportion reported not engaging in enough physical activities [10]. Other risk factors are commonly present; for example, 18% of the population reported that they were smokers [10]. A comparison of mortality due to non-communicable diseases in Saudi Arabia to western European nations shows that death occurs at a younger age among Saudis. If the current trend persists, the percentage of Saudis with noncommunicable diseases is expected to double by the year 2030 [10].

Several screening programs exist in Saudi Arabia including screening programs for CRC and breast cancers [11,12]. While others including cervical, prostate, and lung cancers do not have an established screening program (lung and cervical cancer) or the recommendations for screening are based on shared decision making as in the case of prostate cancer [13-15]. The screening services are freely available in governmental hospitals and primary healthcare centers (PHCs) [11,12]. The process of screening usually begins with a PHC physician recommending or referring a patient for a screening test. Noninvasive tests like fecal occult blood testing (FOBT) for CRC are available at the level of PHCs, while invasive tests or those that require radiological exposure are available at referral hospitals as is the case for total colonoscopy and breast mammogram [11,12]. Additionally, some might be available in mobile clinics in the case of mammograms [12]. CRC screening in PHCs is done through FOBT; if the FOBT is positive then the case will be referred to a referral hospital where further assessment can be done by colonoscopy [11]. Cancer screening uptake in Saudi is reported to be low; uptake of CRC screening was reported to be 5.64% in a sample of the elderly population [16]. For breast and cervical cancers, 92% reported never being screened by mammogram, and only 33.4% went through a Pap smear [17,18]. With regard to prostate cancer, among eligible age groups for screening, 41.7-52.5% reported that they were screened by prostate-specific antigen (PSA) testing, while the percentage increased with higher age groups [19].

In recent years, Saudi Arabia initiated a large-scale program, the National Transformation Program (NTP), as a part of the Kingdom’s Vision 2030; the program includes the cooperation of various governmental bodies to achieve many strategic objectives of Vision 2030. A pillar of Vision 2030 is to have a vital society; this pillar is reflected in the strategic goal of health promotion and the reduction of health risks [3]. The new healthcare model, which is a part of the NTP and is guided by the Saudi Ministry of Health, aims to focus on preventive health and strengthen the role of PHCs [10]. Thus, it is crucial to assess the practices of PHC physicians regarding various cancer screening programs and barriers that might affect the outcome of these preventive programs that are run by or depend on PHC settings in order to overcome these barriers and anticipate them in such activities.

## Materials and methods

Study design and setting

The study followed a cross-sectional design among Ministry of Health PHC physicians. The study was conducted in PHCs in the Al-Qassim region, including all PHCs affiliated with the Ministry of Health.

Participants

Convenience sampling techniques were used. The list of PHCs in the Al-Qassim region was obtained from the Directorate of Health Affairs (Ministry of Health) in Al-Qassim region. Each PHC in major cities was approached by the study investigators, and the study survey was distributed among all the on-duty physicians present during the visits. Additionally, an online survey link was distributed to PHC physicians in Al-Qassim through WhatsApp (WhatsApp LLC, Menlo Park, California, United States) groups to allocate more participants. The inclusion criterion was PHC physicians who work in MOH PHCs in the Al-Qassim region of Saudi Arabia. Interns, pediatricians, and incomplete responses were excluded from the final analysis. PHCs that were dedicated to coronavirus disease 2019 (COVID-19) management during the past year were excluded. The calculated sample size was 208 based on the following assumptions: 95%CI, population size of 450, which represents the number of PHC physicians in the Al-Qassim region, and anticipated percentage frequency of 50%; the sample calculation was done through Epi Info™ [20].

Variables

The study survey was self-administered and made available in English and Arabic languages. It was filled out electronically using an online form on data collectors' tablets when accessible or through an online link when the PHC was not accessible or physicians were not available at the time of the visit. The first page included information on the study purpose, objective, time needed for completion, ethical approval, and the contact details of the supervising ethical committee and study supervisor. Participation was optional.

The study survey was structured into three parts. The first part covered sociodemographic aspects such as age, gender, nationality, and level of education. It also included items related to specialty per the Saudi Committee of Health Specialties (SCHS) as well as job title/classification per SCHS and years of experience ranging from less than five years to more than 15 years. Cancer family history items were included, as well as whether that history was among first-degree relatives. The second part included questions on the clinical practice with regard to the average number of patients seen by the PHC physician in a single day and items on the practice of cancer screening for patients at average risk of colorectal, breast, prostate, lung, and cervical cancers during the last 12 months. The third part covered various barriers to recommending a screening test related to patients (i.e., patients' fear of finding cancer, patients' belief that cancer screening is not effective, and the lack of signs and symptoms of cancer in question). Physicians’ barriers were included as well, including lack of equipment or facility for a screening test, lack of training needed for recommending or conducting a screening test, poor patient compliance, not having enough time to discuss the screening test with the patient, being unaware of cancer screening guidelines, and believing that recommending the screening test is not part of their role as PHC physicians. Barriers related to the system in place included not being common practice for physicians in certain PHCs to recommend or refer patients for screening tests, a shortage of trained healthcare providers who could conduct specific screening tests in the referral hospital, a screening test not being covered by the Ministry of Health, no clear pathway for referral, difficulty in scheduling, and poor feedback on the outcome of the screening test. Different categories of barriers had an option of “others” to account for barriers that were not included. Response to the barriers was categorized on a five-point Likert scale ranging from strongly agree to strongly disagree and was summarized into a three-point scale as agree, neutral, and disagree.

Survey items were based on a review of previous literature and were further validated by three consultants in preventive medicine and public health; they were tested through a pilot study among PHC physicians before the initiation of the study [4,21-23]. Reliability assessment for barriers for screening showed Cronbach's alpha to be 0.81 for system barriers, 0.72 for physicians' barriers, and 0.58 for patients' barriers; the overall Cronbach’s alpha was determined to be 0.838, which confirms that there is a high level of internal consistency and reliability for the questions used in eliciting participants’ responses on what they think to be the barriers to cancer screening recommendations.

Outcome variables

Our primary outcome was the practices of PHC physicians with regard to cancer screening for average-risk patients ("individuals with no significant increase of cancer risk such as predisposition by inheritance, prior diagnosis of pre-cancerous conditions or cancers, and without associated diseases that are known to predispose individuals to more cancer risk") [24] for common cancers including CRC and breast, prostate, lung, and cervical cancers during a month. The secondary outcome was to identify various barriers to recommending a screening test; these barriers included three categories: patient-related, physician-related, and system-related barriers as perceived by PHC physicians.

Statistical analysis

Data were collected from study respondents and entered into a Microsoft Excel spreadsheet (Microsoft Corporation, Redmond, Washington, United States) within which cleaning and validation of the entered data took place. Following cleaning, the valid data were imported into IBM SPSS Statistics for Windows, Version 22.0 (Released 2013; IBM Corp., Armonk, New York, United States) for data analysis. The focus of the statistical analysis was to assess the frequency of cancer screening among the study respondents and identify the common barriers that influenced physicians to recommend cancer screening tests. Descriptive analysis was first carried out with the results presented in frequency tables showing the distribution of categorical variables in numbers and percentages while continuous variables such as age were presented as mean and standard deviation. Barriers to screening were presented in bar charts as percentages. Logistic regression analysis was done to identify sociodemographic factors that influence cancer screening recommendations among physicians on a cancer-type basis. The level of significance was set at <0.05.

Ethical considerations

Research investigators obtained ethical approval from the regional ethical committee of the Directorate of Health Affairs in the Al-Qassim region (approval number: 1443-598106). The data collection process followed the ethical committee guidelines for the protection of human subjects concerning their safety and privacy. Participation was voluntary and on the first page of the survey, it was asked if the respondent agreed to participate. Choosing the option "Yes" was considered consent for participation.

## Results

Participants' sociodemographic and clinical experience

Descriptive analyses of the collected data (n=141) showed that the larger proportion of study respondents (Table 1) were males (60.3%) and the mean age of the entire population was 35.7 ± 8.3 years, with a range of 25-60 years. Many of the respondents worked in Buraydah (36.9%), Alrass (17.0%), or Unaizah (13.5%). Regarding nationality, non-Saudi physicians were more prominent, with a proportion of 58.9%. The majority of the study population had a bachelor’s degree in the form of an MBBS (Bachelor of Medicine, Bachelor of Surgery) or an MD (Doctor of Medicine) degree (74.5%), followed by a Ph.D. degree or board certification (12.8%).

**Table 1:** General characteristics of the study respondents (n = 141) SD: Standard deviation; SCHS: Saudi Committee for Health Specialties

Variable	Values	Frequency	Percent
Age (Mean ± SD, years)		35.7 ± 8.3	
Gender	Female	56	39.7
	Male	85	60.3
Location of work	Buraydah	52	36.9
	Alrass	24	17.0
	Unaizah	19	13.5
	Al Mithnab	5	3.5
	Others	41	29.1
Nationality	Saudi	58	41.1
	Non-Saudi	83	58.9
Highest level of education	Bachelor	105	74.5
	Diploma	6	4.3
	Master’s	12	8.5
	PhD or board cert.	18	12.8
Specialty as per SCHS	General practice	83	58.9
	Family medicine	43	30.5
	Internal medicine	12	8.5
	Obstetrics/Gynecology	3	2.1
Job title as per SCHS	Consultant	7	5.0
	Registrar/senior Registrar	18	12.8
	Resident	116	82.3
Years of experience	<5 years	55	39.0
	5-9 years	31	22.0
	10-15 years	31	22.0
	>15 years	24	17.0
No. of patients seen per day	1-5 patients	3	2.1
	6-10 patients	15	10.6
	11-20 patients	40	28.4
	>20 patients	83	58.9
Family history of cancer	No	112	79.4
	Yes	29	20.6
Cancer in first-degree relative (n = 29)	No	16	55.1
	Yes	13	44.8

The most common specialty was General Practice, which constituted 58.9% of the respondents. Family medicine (30.5%) and Internal Medicine (8.5%) were also common specialties among the respondents. The majority of the respondents were residents (82.3%) followed by registrar/senior registrars (12.8%) and consultants (5%). Physicians with less than five years of experience were the most common (39.0%), followed by those with 10-15 years of experience (22.0%) and those with five to nine years of experience (22.0%) as well. Many of the respondents saw upwards of an average of 11 or more patients per day, with 58.9% seeing even more than 20 patients per day and 28.4% seeing between 11 and 20 patients daily. Only about one-fifth of the respondents reported that they had a family history of cancer (20.6%) and about half reported having this in a first-degree relative (44.8%).

Physicians’ experience and practice of cancer screening

Going further to look into the respondents' experience with recommending cancer screening (Table 2), more than three-quarters of the physicians had referred a patient for cancer screening at some point in their experience (79.4%). Of this, the rate of recommending cancer screening varied by the type of cancer, with screening for CRC being the most prominent (64.5%), followed by breast (51.8%), prostate (16.3%), lung (12.1%), and cervical (11.3%) cancers. When split by specialty, general practitioners appeared to be the ones who most commonly referred patients for screening for all types of cancers, except in the case of cervical cancer, which was dominated by family medicine specialists (62.5%). The majority of the referrals for CRC (58.2%), breast cancer (54.8%), prostate cancer (52.2%), and lung cancer (64.7%) were carried out by general practitioners.

**Table 2:** Percentages of doctors who recommend screening for various types of cancers (n = 141) GP: General practitioner; Obs/Gyn: Obstetrics and Gynecology

Cancer type	Referred patients for screening n (%)				
		GP	Family medicine	Internal medicine	Obs/Gyn
Colorectal	91(64.5%)	53 (58.2%)	33 (36.3%)	5(5.5%)	0 (0.0%)
Breast	73(51.8%)	40(54.8%)	28 (38.4%)	4 (5.5%)	1 (1.4%)
Prostate	23 (16.3%)	12 (52.2%)	8 (34.8%)	3 (13.0%)	0(0.0%)
Lung	17 (12.1%)	11 (64.7%)	3 (17.6%)	3 (17.6%)	0 (0.0%)
Cervical	16 (11.3%)	3 (18.8%)	10 (62.5%)	1 (6.3%)	2 (12.5%)

Barriers influencing physician decisions in referring patients for cancer screening

**Figure 1:**
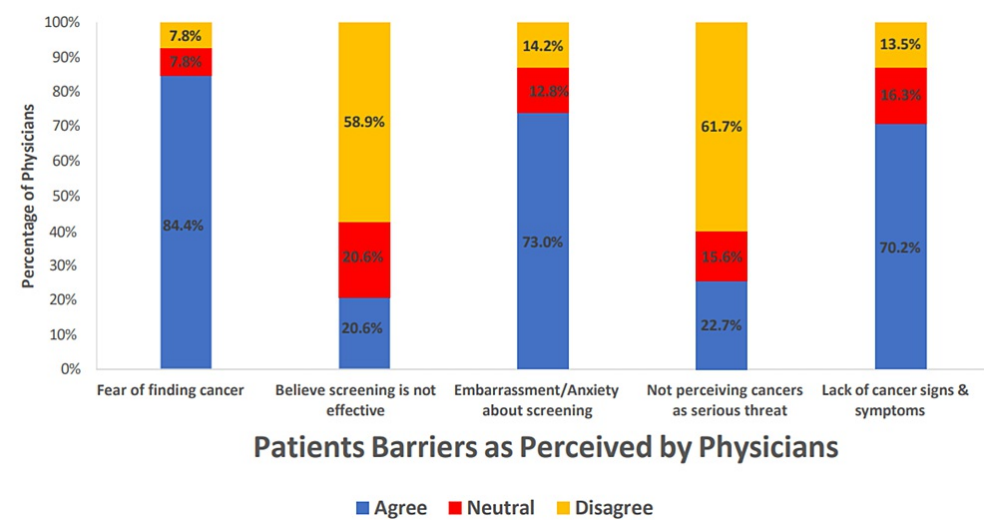
Bar chart showing patient barriers to screening recommendation among physicians

**Figure 2:**
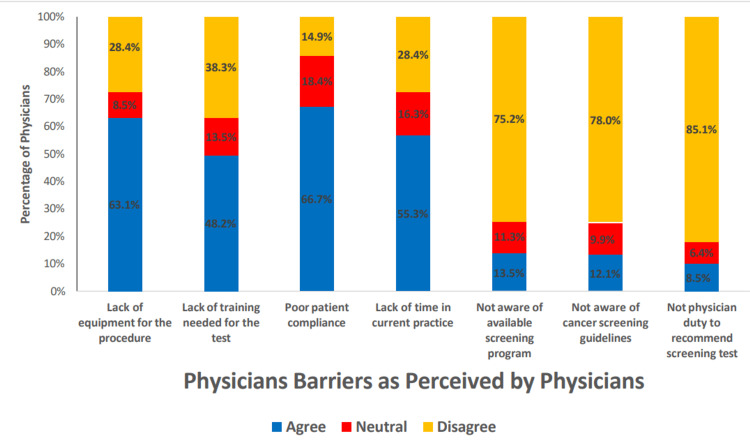
Bar chart showing physician barriers to cancer screening recommendation

**Figure 3:**
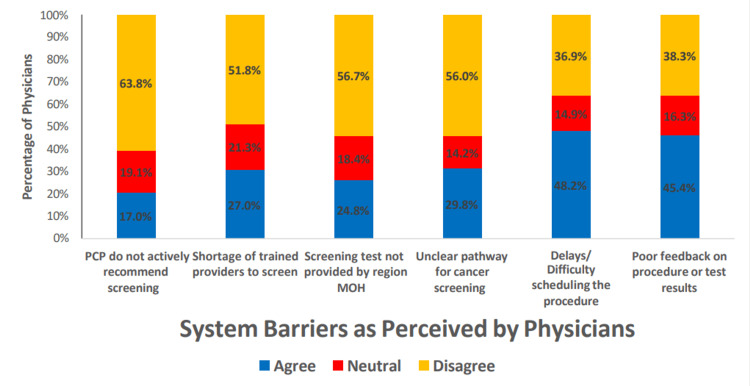
Bar chart showing physician barriers to cancer screening recommendation PCP: Primary care physician; MOH: Ministry of Health

Several barriers were identified as important among physicians deciding to refer patients and patients refusing to accept a recommended cancer screening test or procedure (Figures 1-3). Within the category of patient barriers, the most prominent was fear of finding cancer, with more than 80% of the physicians agreeing or strongly agreeing with this statement. Two other major patient barriers were also reported: embarrassment or anxiety about the screening test (73.0%) and the lack of signs and symptoms related to the cancer of interest, with about 70.2% of the respondents agreeing that this was a common reason for refusal to accept a cancer screening test or procedure. Physician barriers were also common; the most frequent included poor patient compliance (66.7%), lack of equipment for the procedure (63.1%), lack of time in current practice (55.3%), and lack of training needed for the test (48.2%). The respondents disagreed or strongly disagreed that recommending cancer screening is not the duty of the physician (85.1%), non-awareness of cancer screening guidelines (78.0%), and non-awareness of available screening programs (75.2%) were reasons for not recommending patients for cancer screening.

Physician responses to systemic barriers to cancer screening appeared mixed, with about one-sixth to one-fifth of the respondents preferring to stay neutral across the range of responses. The most common barriers that the physicians thought affected cancer screening included delays and/or difficulty in scheduling the procedure (48.2%), poor feedback on the procedure or test results (45.4%), and a non-clear pathway for cancer screening (29.8%).

Factors influencing recommendation for cancer screening tests/procedures

Adjusted for all other factors, gender influenced cancer screening recommendations for breast, prostate, lung, and cervical cancers as shown in Table 3. Male physicians were less likely to recommend patients for breast (0.10, 95%CI 0.04-0.23, p < 0.001) and cervical (0.26, 95%CI 0.08-0.78, p = 0.017) cancer screening. On the other hand, however, male physicians were 3.74 times more likely to recommend for prostate cancer screening (95%CI 1.20-11.68, p = 0.023) and 5.79 times more likely to request for lung cancer screening (95%CI 1.27-26.39, p = 0.023).

**Table 3:** Logistic regression table showing factors influencing screening recommendations for different types of cancers (n = 141). *p-value is less than 0.05; **p-value is less than 0.001; ^a^p value is >0.05 but <0.1 Reference values: 1-Females, 2-Non-Saudi, 3-Bachelors degree, 4-Non-GP specialties (family medicine, internal medicine, and O&G), 5-Residents, 6-Less than five years, 7-Seeing 1-10 patients, 8-No family history of cancer GP: General practitioner; O&G: Obstetrics and Gynecology

Variable	Colorectal	Breast	Prostate	Lung	Cervical	
Age	1.01 (0.96-1.05)	1.00 (0.96-1.04)	1.03 (0.98-1.09)	1.03 (0.97-1.09)	1.04 (0.98-1.11)	
Gender^1^	0.89 (0.44-1.82)	0.10 (0.04-0.23)**	3.74 (1.20-11.68)*	5.79 (1.27-26.39)*	0.26 (0.08-0.78)*	
Nationality^2^	1.22 (0.60-2.48)	0.55 (0.28-1.09)^a^	1.12 (0.45-2.77)	0.76 (0.26-2.17)	0.62 (0.20-1.88)	
Education^3^	1.04 (0.47-2.29)	0.95 (0.44-2.02)	0.74 (0.28-1.99)	1.13 (0.34-3.72)	0.21 (0.07-0.63)*	
Specialty^4^	0.93 (0.46-1.88)	0.70 (0.36-1.38)	0.72 (0.29-1.77)	1.32 (0.46-3.81)	0.13 (0.04-0.48)*	
Job level^5^	2.54 (0.89-7.24)^a^	2.85 (1.11-7.35)*	2.43 (0.88-6.74)	0.59 (0.13-2.74)	6.35 (2.10-19.19)*	
Years of experience^6^	0.94 (0.46-1.90)	0.74 (0.38-1.47)	1.25 (0.51-3.08)	0.83 (0.29-2.40)	0.48 (0.15-1.58)	
Number of patients^7^	0.39 (0.14-1.05)^a^	0.55 (0.20-1.51)	1.56 (0.46-5.27)	1.56 (0.40-6.06)	0.42 (0.05-3.42)	
Family history^8^	0.73 (0.32-1.68)	0.59 (0.26-1.35)	2.46 (0.93-6.56)	0.48 (0.10-2.23)	0.52 (0.11-2.42)	

Respondents' level of education was a factor only for cervical cancer as those with a bachelor's degree were 4.76 times more likely to request cervical cancer screening (95%CI 0.07-0.63, p = 0.005) compared to those with a diploma, master's, or Ph.D. degree. Similarly, physicians who work in non-general practice specialties (as opposed to those who work in general practice) were 7.16 times more likely to recommend cervical cancer screening (95%CI 0.04-0.48, p = 0.002). Senior physicians such as registrars/senior registrars and consultants were 2.85 times more likely to request or recommend a patient for breast cancer screening (95%CI 1.11-7.35, p = 0.030) and 6.35 times more likely to recommend for cervical cancer screening (95%CI 2.10-19.19, p = 0.001).

Factors such as the nationality of the participants, number of years of experience, number of patients seen on a daily basis, and family history of cancer did not have any statistically significant influence on cancer screening recommendations across the six types of cancers investigated (p > 0.05).

## Discussion

Screening and early identification of cancer are the most effective approaches to lower cancer morbidity and mortality. Specific cancers may be prevented or identified at their early stage, when treatments are most successful, to prevent further progression to advanced stages [25]. Screening procedures have been proven to be influenced by physicians' recommendations and patients' knowledge, attitudes, and perceptions [26,27]. The current study findings showed that more than three-fourths of the physicians referred a patient for cancer screening at some point in their clinical practice. Our findings regard cancer screening are higher for CRC in the Al-Qassim region of Saudi Arabia at 64.5% vs 42% in Norway; this might be due to the fact that the project for CRC screening has been initiated by the Ministry of Health recently, while in Norway, the program was not initiated at the time of the survey [4]. However, the figure is lower than CRC screening recommendation rates in Riyadh (92%) [28]. PHC physicians in Al-Qassim recommend their patients for mammograms less than their counterparts in Norway (51.8% vs 89%); this might be due to the fact that the breast cancer program in Saudi Arabia encounters some difficulty in terms of clarity in seeking the service [3]. associated with a personal fear of doctors, hospitals, and the consequences of finding cancer as seen in our study and another one in the Eastern region [29]. Another study, done by Alshahrani et al. among female patients in PHCs in Najran, Saudi Arabia, reported that only 8.8% received information about breast cancer screening through physicians [30]. Lung cancer screening is low in both our study (12.1%) and Norway (17%) [4], as it is not part of established screening programs in the two countries. The highest difference is noted with cervical cancer screening with 94% in Norway vs 11.3% found in our study which can be attributed to the established program in Norway which is not the case in Saudi Arabia although some studies anticipated the cervical cancer incidence to rise in the kingdom [4,14]. For prostate cancer, the screening recommendations are conflicting and based on decision-sharing with the patient [8].

Similar to the observation of the study by Abdel-Aziz et al. conducted to identify the patients' perceived barrier to breast cancer screening [29], the most agreed-upon barrier by the participants in the current study was the ''fear of finding cancer'' after the screening. Meanwhile, the most agreed-upon physicians’ perceived barrier was poor patient compliance, followed by a lack of equipment for the screening procedure as observed in another study [31]. It is reported that insufficient knowledge and lack of proper advice from physicians were identified as significant barriers to cancer screening practices among patients [32]. A similar study among family physicians in Riyadh also reported that lack of patient awareness was the most prominent barrier for patients as perceived by physicians to undergo colorectal cancer screening. The study found that barriers were reported to be significantly higher among physicians not practicing cancer screening than among practicing physicians [33]. Our study's most identified system barriers were delays and/or difficulty in scheduling the procedure and poor feedback on the procedure or test results as in the study by Alghamdi et al. [31]. A physician's nonprofessional experience with cancer, such as family members having cancer, can impact their screening practice patterns. A national survey done in the United States among primary care physicians reported that those with nonprofessional cancer experience were more likely to recommend cancer screening than those without this experience [34]; however, in our study sample, this did not affect cancer screening practices. In our study, it was found that male physicians' were less like to recommend screening for breast and cervix cancer screening compared to female physicians; at the same time, male physicians were more likely to recommend lung and prostate cancer screening than females. This finding is in line with the findings of Bringedal et al., who reported that male physicians were more likely to consider mammogram screening as non-necessary and a waste of resources [4,35]. However, there have been conflicting reports in which male physicians were more likely to recommend cancer screening every one or two years, whereas female physicians advised comparatively fewer screenings or none at all [26]. The current study findings also showed that senior physicians such as senior registrars and consultants were more likely to request or recommend a patient for breast and cervical cancer screening. One possible explanation for this higher number of recommendations could be the higher number of patients they routinely face and the experiences they have acquired throughout their training and practice.

Evidence-based guidelines for healthcare professionals have been produced to decrease heterogeneity and enhance the usage of relevant cancer screening methods. For many patients, getting a physician's advice to undergo cancer screening is critical in determining their decision to go through with the screening tests [36]. Our findings provide a piece of important information as guidelines and programs regarding cancer screening continue to evolve; they underscore the need to delineate barriers to and facilitators of implementation in clinical practice.

Limitations

Some of the limitations of this study should be explained before the findings are extrapolated. First, the study reported screening based on self-reported cancer screening practices, which might overestimate the actual practices as well as be prone to recall bias. Therefore, future studies might need to follow the actual implementation of these screening recommendations based on patients’ health records. When the barriers to screening were assessed, they were not tailored to specific screening tests or certain types of cancer; instead, they were assessed in general for any cancer. Therefore, we cannot decide if that barrier is specific to certain cancer or not, which might limit the ability to draw a conclusion about barriers for specific cancer screening or even specific screening tests. The study was conducted in the Al-Qassim region of Saudi Arabia. The sample size might limit the power of the study to evaluate the associated factors with screening practices as well the generalizability of the study’s findings, but can provide a prospect on the practices of PHC physicians in the region but not to those in the other provinces of the kingdom, nor to private clinics or PHCs run by entities other than the Ministry of Health. However, the study provides insight into practices regarding cancer screening with established screening programs, i.e., breast cancer versus others with other with no established program, such as cervical cancer, as well barriers to practice cancer screening as perceived by PHC physicians.

## Conclusions

Screenings for CRC and breast cancer were the commonly recommended tests. Patients' fear of finding cancer, poor patient compliance, and delays and/or difficulty in scheduling the procedures were the commonly identified barriers as perceived by physicians that influenced physician decisions in referring patients for cancer screening. Our findings suggest that cancer screening rates may be improved by educating individuals for benefit of early detection of cancers and providing assurance for them with regard to the availability of effective treatments. More research is needed on ways to overcome the obstacles encountered by physicians and the outcomes of these measures with regard to improved screening practices.
